# The effectiveness of nutrition education for overweight/obese mothers with stunted children (NEO-MOM) in reducing the double burden of malnutrition in Indonesia: study protocol for a randomized controlled trial

**DOI:** 10.1186/s12889-016-3155-1

**Published:** 2016-06-08

**Authors:** Trias Mahmudiono, Triska Susila Nindya, Dini Ririn Andrias, Hario Megatsari, Richard R. Rosenkranz

**Affiliations:** Department of Nutrition – Faculty of Public Health, Universitas Airlangga, Jl. Mulyorejo Kampus C, Surabaya, 60115 Indonesia; Department of Health Promotion and Behavioral Education – Faculty of Public Health, Universitas Airlangga, Jl. Mulyorejo Kampus C, Surabaya, 60115 Indonesia; Department of Food, Nutrition, Dietetics & Health, Kansas State University, Manhattan, KS 66506 USA

**Keywords:** Nutrition education, Double burden of malnutrition, Indonesia

## Abstract

**Background:**

Nutrition transition in developing countries were induced by rapid changes in food patterns and nutrient intake when populations adopt modern lifestyles during economic and social development, urbanization and acculturation. Consequently, these countries suffer from the double burden of malnutrition, consisting of unresolved undernutrition and the rise of overweight/obesity. The prevalence of the double burden of malnutrition tends to be highest for moderate levels (third quintile) of socioeconomic status. Evidence suggests that modifiable factors such as intra-household food distribution and dietary diversity are associated with the double burden of malnutrition, given household food security. This article describes the study protocol of a behaviorally based nutrition education intervention for overweight/obese mothers with stunted children (NEO-MOM) in reducing the double burden of malnutrition.

**Methods:**

NEO-MOM is a randomized controlled trial with a three-month behavioral intervention for households involving pairs of 72 stunted children aged 2–5 years old and overweight/obese mothers (SCOWT) in urban Indonesia. The SCOWT pairs were randomly assigned to either an intervention group or to a comparison group that received usual care plus printed educational materials. The intervention consisted of six classroom sessions on nutrition education and home visits performed by trained community health workers using a motivational interviewing approach. The primary outcomes of this study are the prevalence of double burden of malnutrition as measured in SCOWT, child’s height-for-age z-score (HAZ) and maternal body mass index (BMI).

**Discussion:**

Because previous studies are mainly observational in nature, this study advances understanding of the double burden of malnutrition through a fully powered randomized controlled trial. The intervention assists participants in self-administered goal setting to improve diet and child feeding behaviors by improving self-efficacy. Maternal self-efficacy may be enhanced through vicarious and active mastery of experiences gained during six sessions of nutrition education and verbal persuasion during home visits.

**Trial registration:**

The Universal Trial Number (UTN) for this study is U1111-1175-5834. This trial was registered in the Australian New Zealand Clinical Trials Registry (ANZCTR) and is allocated the registration number: ACTRN12615001243505 on 12 November 2015.

## Background

### Double burden of malnutrition

Children of the world still suffer from malnutrition, and more than 25 % of under-five children in developing countries are stunted [1]. Stunted children do not grow well, and they are shorter in stature than normal children of their age. Childhood stunting is associated with suboptimal brain development, which leads to impaired cognitive ability and school performance, and reduces earning potential later in life [2]. Acknowledging the detrimental consequences of child stunting, the World Health Organization (WHO) and its member countries have pledged to achieve a 40 % reduction by 2025 through the Scaling up Nutrition (SUN) program [2].

While the battle with stunting is far from over, developing countries have been hampered by another nutrition problem. In the mid-1990s, Popkin [3] observed that the phenomenon of coexistence between overweight and obesity and undernutrition mostly occurred in developing countries. Since then, the double burden of malnutrition has raised public health concerns regarding its consequences. First, there is, concern that the burden of disease may increase as a result of vitamin, mineral and protein deficiencies brought about by undernutrition. Overnutrition increases the risk of noncommunicable diseases (NCD), including obesity, hypertension, type 2 diabetes, and cardiovascular diseases. The presence of NCDs accounted for 80 % of the total disease mortality burden in developing countries, with an estimated $84 billion of economic production lost due to heart disease, stroke, and diabetes alone [4]. Hence, the presence of double burden of malnutrition poses an additional burden on the already inadequate and overextended health budget in developing countries [5].

Most developing countries experience nutrition transition, or rapid changes in dietary patterns and nutrient intakes, when populations adopt modern lifestyles during economic and social development, urbanization and acculturation [3]. Popkin [6] argued that the double burden of malnutrition in one household is related to urbanization. With urbanization, household incomes increase and food becomes more available in terms of quantity, but not quality [7]. Available food is high in energy, but low in micronutrients and protein that affect child growth, especially height. Receiving insufficient micronutrients and protein in the first two years of life, increases the child’s risk of being stunted [2]. Mothers that consume high energy food accompanied by low physical activity, are at increased risk of overweight and obesity. Indonesia, the fourth most populous country in the world, has suffered from the double burden of malnutrition. The prevalence of child stunting in Indonesia was 36.8, 35.6 and 37.2 % in 2007, 2010 and 2013, respectively [8], while the prevalence of obesity (based on Asian cut-off BMI > 27) among women (aged > 18 years old) has continued to increase. In 2007 the obesity prevalence among women was only 13.9 %; in 2010 it was 15.5 % and in 2013 it was 32.9 % [8]. Some have suggested that East Java Province is a microcosm of Indonesia in terms of achievement in child health outcomes in Indonesia. The prevalence of child stunting in East Java was similar to the national figure of around 35 % [8]. A study in rural Indonesia showed that the prevalence of the coexistence of child undernutrition that includes stunting and maternal overweight/obesity (SCOWT) was 11 % [9].

Evidence shows that food insecurity is one of the risk factors for child stunting, but there is currently little evidence that food insecurity is risk factor for double burden of malnutrition [10]. A cross sectional study in rural Indonesia demonstrated that higher intakes of animal products was protective against SCOWT [11], but the lack of animal protein intake was more indicative of dietary diversity than a valid indicator for food insecurity. A previous study revealed the double burden of malnutrition to be most prevalent, at 22.7 %, among the middle (third) quintile of socioeconomic status (SES) in a Guatemalan population [10]. This evidence suggests that at the household level, in the absence of food insecurity and economic deprivation, modifiable factors such as food distribution and dietary diversity were associated with the high prevalence of double burden of malnutrition in this third quintile group, relative to the others. We hypothesize that stunted children in households that suffer from the double burden of malnutrition are less likely to be facing food insecurity. Evidence showed that strategies to reduce overweight and obesity should emphasize recommending increased fruit and vegetable consumption with explicitly combining this approach with other efforts [12] such as increasing physical activity [13].

We aim to target the modifiable behaviors related to the double burden of malnutrition in an urban setting in Indonesia through an intervention offering nutrition education for overweight/obese mothers with stunted children (NEO-MOM). This article describes the rationale and methods for the randomized controlled trial conducted in urban Indonesia to reduce the prevalence of double burden of malnutrition as measured in SCOWT pairs.

## Methods/Design

**Fig. 1:**
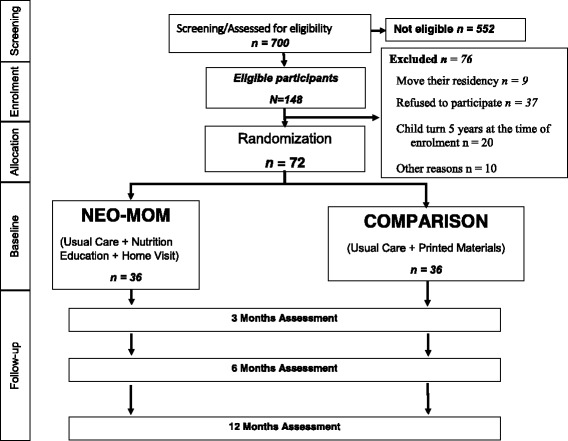
Adapted CONSORT diagram of the study

This RCT was a superiority trial consisting of 2-arm parallel groups (an intervention group and a comparison group that received usual care along with printed educational materials). A three-month behavioral intervention was delivered to the two groups made up of households with stunted children aged 2 to 5 years and overweight/obese mother (SCOWT pairs). After an initial screening procedure, the 72 women with stunted children who consented to participate were randomly assigned to either the intervention or comparison group. The study employed parallel assignment with the two groups of participants receiving different interventions during the same time span. The two groups — the enhanced behavior change intervention group (NEO-MOM) and a comparison group receiving printed materials (PRINT) — were each made up of 36 overweight/obese mothers with a stunted child. Baseline data was gathered from participants in their homes at the beginning of the study. Another visit to collect post-intervention data followed the three-month intervention. The adapted CONSORT diagram in Fig. 1 shows progression through the study for individual participants.

### Objective and hypothesis

The objective of the RCT was to evaluate the effectiveness of a behaviorally based nutrition education (NEO-MOM) intervention in reducing the prevalence of household double burden of malnutrition as indicated by stunted child and overweight/obese mother (SCOWT) pairs.

#### Primary hypothesis

The NEO-MOM intervention, consisting of six sessions of behaviorally based nutrition education and home visits, will be more effective in reducing SCOWT prevalence in the intervention group, relative to the comparison group.

#### Secondary hypothesis

We hypothesized that after six sessions of behaviorally based nutrition education (NEO-MOM) accompanied by biweekly home visits, relative to the comparison group, mothers in the intervention group would:

1. Show a greater increase in average maternal fruit and vegetables intake and average child animal protein intake.
2. Show a greater increase in maternal self-efficacy for fruit and vegetable intake, as well as improved child-feeding behavior for protein intake.
3. Show a greater increase in maternal physical activity (average number of steps per day assessed via pedometer).
4. Show larger reductions in maternal sedentary time.

### Ethical approval

The Institutional Review Board (IRB) at Kansas State University approved the trial (reference or proposal number: 7894). The screening portion was also approved by the IRB at Kansas State University (reference or proposal number: 7646). In addition, this trial is approved by the Surabaya City Review Board (Bakesbangpol No: 1366/LIT/2015) in Indonesia. The main ethical consideration was to ensure that risk of harm to participants was minimized and that they were fully informed of any risk. Religious and cultural sensitivities were taken into account when obtaining informed consent. Recruitment and informed consent were handled so potential participants were not pressured to participate, and confidentiality was preserved. All participant data was stored using code system and electronic data remains password protected.

We obtained informed consent during monthly community health post meeting (*posyandu*), where mothers brought children under five years of age for growth monitoring. Research assistants invited potential participants to meet in a separate room, giving them verbal and written information about the study and at least one week to think about their participation. Participants were free to withdraw from the study at any time without negative consequences.

A copy of participant informed consent will remain on file for 12 months after the completion of the study. Personal data with participant identification will be destroyed after 3 years following completion with other records archived for five years before being destroyed. The Universal Trial Number (UTN) for this study is U1111-1175-5834. This trial is registered in the Australian New Zealand Clinical Trials Registry (ANZCTR) and is allocated the registration number: ACTRN12615001243505. The ANZCTR is recognized as an ICMJE (the International Committee of Medical Journal Editors) acceptable registry and a Primary Registry in the WHO registry network.

### Setting

The study is set in urban city of Surabaya, Indonesia. Surabaya is the second largest city in Indonesia with more than 3.1 million inhabitants and 5.6 million in the metropolitan area [14]. Popkin [3] has argued that urbanization is one of the driving forces for the double burden of malnutrition, and that Surabaya is a prime setting for studying the phenomenon of nutrition transition and the double burden of malnutrition. Surabaya has a big port and industries that make it a melting pot for urbanization from the eastern part of Indonesia. Surabaya was chosen as the setting of this study because it spans the range of population densities, urbanization, and socioeconomic profiles. Because Indonesia is an archipelago country, recruiting participants from every major city on each island would have been cost prohibitive. A limitation, however, is that Surabaya may not be representative of urban cities in Indonesia and may not address health and nutrition inequalities across islands. Many health and nutrition inequalities exist within Surabaya City that mirror other urban settings in Indonesia [15].

### Target population

The sample was drawn from community health posts *(“posyandu”)* [16] listing more than 50 mothers with children under the age of five. Informed consent was gathered from all participants before undergoing screening to validate their eligibility for participation. The case definition included overweight/obese mothers (BMI > 25) with stunted children (HAZ < -2). A BMI cut-off point of >25 kg/m2 was used to determine maternal overweight and obesity, while child stunting was defined as HAZ < -2, according to the child growth standard from the WHO-Anthro 2005. Repeated anthropometric measurement for both mothers and children are performed by trained research assistance in *posyandu*. A third measurement was initiated when the discrepancy between the first and the second measurement was greater than 1 %. An average of anthropometric measurements was used to determine eligibility. Following screening via anthropometric measurement, those eligible were invited to participate in the study.

### Inclusion and exclusion criteria

Inclusion criteria required participants to be fluent in conventional Bahasa Indonesia language, a permanent resident planning to stay in Surabaya City for at least six months, the mother of a stunted child under age five, and overweight/obese. In addition, the child had to be registered in *posyandu* and receiving food supplementation from the government. Participants were excluded in cases where either the mother or the child had physical disability, mother was participating in a weight loss program or deliberately fasting due to a spiritual deed; mother was pregnant; or the child had been diagnosed with serious medical problems.

### Power calculation

The power calculation of the main outcome variable was based on a previous RCT in Bangladesh that explored effects of a similar three-month nutrition education intervention with complementary feeding on child’s height gain [17, 18]. Although the study did not directly provide complementary feeding, one of the inclusion criteria was that participants would be eligible to take part in a complementary feeding program administered by the government of Indonesia. The effect size of providing complementary food and intensive nutrition education on height gain (cm) in Bangladesh was 0.80 (95 % CI = 0.007–1.53). We selected this fairly large effect size of 0.8 expressed as mean difference in child height, which translates to an ability to detect a difference between two groups of 0.80 cm in child height at a three-month follow-up. The study has a 90 % power to detect modest changes at the individual level that would have an important impact if occurring at the population level. A minimum total sample size of 66 was needed to detect these differences in our primary hypothesis, with two-tailed alpha of 0.05. Assuming a dropout rate of 9 %, the total sample size of 72 was required (36 in the intervention group and 36 in the comparison group/usual care).

### Randomization and allocation concealment

Randomization of participants was performed using a computer to generate random numbers. After assignment of random numbers to the 72 consenting participants, the list was sorted based on the random number. Thirty-six participants in the upper rank were assigned to the intervention group (NEO-MOM) and the remaining 36 participants were assigned to the comparison group. Because of the nature of the intervention, blinding for both researchers and participants was not possible. The assessor was blind to the intervention condition for baseline and evaluation data collection.

### Behavioral based nutrition education

**Fig. 2:**
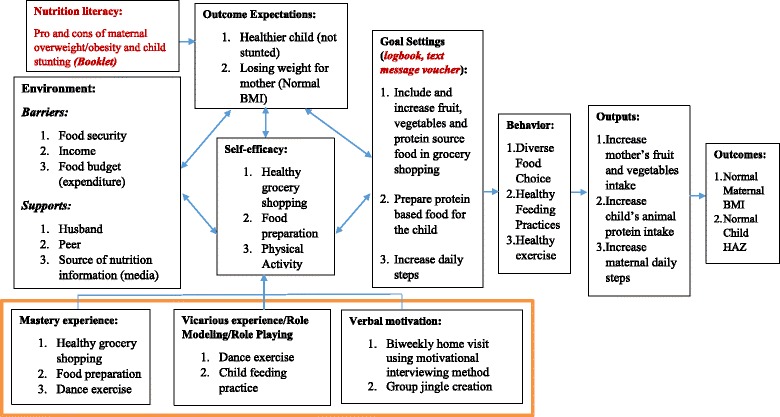
Construct of NEO-MOM Intervention Based on Social Cognitive Theory

Social Cognitive Theory (SCT) was the underlying framework of the study, which focuses on behavior change through reciprocal determinism that incorporates interplay of person, environment and behavior [19]. Bauman, Sallis, Dzewaltowski, and Owen [20] argue that mediating factors lie in the behavior change pathway. In the present study, maternal behavioral change related to overweight/obesity may be influenced by self-efficacy for including more fruits and vegetables in the family menu, and for including more animal protein in their children’s diet for improved iron intake and growth. The behavioral mediators are a part of the personal component in SCT’s reciprocal determinism. The core concept of SCT is the importance of others in shaping people’s behavior. Nutrition education materials, including booklets for all participants, were developed based on the constructs of Social Cognitive Theory (SCT) (Fig. 2). This study addressed several means of enhancing maternal self-efficacy such as mastery of making a healthy menu, peer group modeling of child feeding practices or engaging physical activity and inducing desire to include fruits and vegetables in daily meals.

Because the problem of maternal-child double burden of malnutrition includes two opposing nutritional problems, the target behaviors for this project addressed both childhood stunting and maternal overweight/obesity. For mothers, the target behavior was consumption of fruits and vegetables, which both play a critical role in efforts to prevent overweight/obesity. For the child, the target behavior was increased consumption of animal protein as well as fruit and vegetables.

Manuals published in Bahasa Indonesia or in English in the last five years were identified to improve maternal fruit and vegetables intake as well as to increase child intake of animal protein. Both peer-reviewed articles and gray literature were located and translated into Bahasa Indonesia. Manuals were drafted and refined through a lengthy review process and will be used as the printed educational materials for the intervention.

### Training community health workers

One month before the intervention, community health workers received training on motivational interviewing, a collaborative, goal-oriented style of communication commonly used to promote behavioral change in health care [21]. It is designed to strengthened personal motivation for and commitment to a specific goal by eliciting and exploring the person’s own reason for change within an atmosphere of acceptance and compassion. The core skills of motivational interviewing consist of open questions, affirmations, reflections and summaries. Community health workers will use motivational interviewing during biweekly home visits in the intervention group to give verbal encouragement and find ways to overcome potential barriers for mothers to achieve their goals. The training program took a full day to complete, and was led by study investigators. The training involved a combination of didactic learning, role-playing, and case study discussion.

### Intervention

All participants (NEO-MOM and PRINT Group) received six sets of educational materials in the form of booklets that described strategies to improve the health of stunted children and overweight mothers in alignment with constructs of Bandura’s Social Cognitive Theory (SCT). In addition, mothers were provided with grocery vouchers after consenting to participate in the study during the baseline and evaluation phase. The voucher is worth about USD $1.50, or about IDR Rp. 30,000, and can only be used in designated grocery stores to buy foods and not for alcohol or tobacco.

The intervention group or NEO-MOM group received six sessions of behaviorally based nutrition education focusing on healthy food choice and child feeding practices, along with six sessions of motivational interviewing provided through home visits by a trained community health worker. Mothers were given a biweekly grocery voucher every time they attend a nutrition education session as an appreciation for their time and commitment to the study. The session was conducted on a biweekly basis. In total, the intervention spanned three months. The approximate duration of each nutrition education session was 100 min, consisting of a 50-min class session, followed by a hands-on activity lasting 50 min. The session was administered to mothers with children being allowed to attend and sit with their mothers. Research assistants and community health workers were present during the session to help handle the children when necessary. During the nutrition education sessions, hands-on activities were provided to help mothers improve their self-efficacy toward dietary diversity, healthy eating, and child feeding. These activities were designed to enable mothers to cope with basic household and environmental obstacles. The content of the six sessions of nutrition education are outlined in Table 1.

**Table 1:** Intervention Components of NEO-MOM

Construct of Social Cognitive Theory	Behavior mechanism impacted	Session	Intervention components
Provide information on health risk of maternal overweight/obesity and child stunting	Mechanisms affecting belief formation/cognitive mechanisms toward maternal nutrition literacy	Session 1	50-min nutrition education class on the introduction on the double burden of malnutrition, especially consequences and management of overweight/obesity and child stunting.
Outcome expectation	By improving maternal nutrition literacy, mothers might expect a healthier child (their child not stunted) and improved maternal nutritional status (not overweight/obese).	Session 3	50-min nutrition education class on Indonesian balanced diet and Indonesian version of MyPlate followed by healthy behavior message to improve maternal fruit and vegetable consumption, serve more animal protein to their child and increase maternal daily steps.
Environment (food access, peer support)	With supportive environment, it will be easier to perform the intended healthy behavior.	Session 1–6	Mothers in intervention group gathered every two weeks during nutrition education class to provide bonding and peer support. Access to food increased by distributing a grocery voucher every time mothers in the intervention group attended the nutrition education session.
Mastery experience	Performing intended behavior during nutrition education class or during the hands-on activities session will improve maternal self-efficacy for performing the following behaviors: 1. Healthy grocery shopping 2. Food preparation 3. Physical activity	Session 2 Session 1–6 Session 3 Session 4	50-min nutrition education class on healthy grocery shopping followed by a 30-min mock grocery shopping session. 20-min dance session 30-min making menu for children under five years old with emphasis on including animal protein (chicken liver, catfish or eggs). 30-min nutrition education class on healthy cooking methods followed by a 60-min cooking demonstration.
Goal setting	Assisted planning and goal setting will make behavior change perceived as attainable by mothers.	Session 1 Session 6	30-min hands-on experience on goal setting to improve physical activity (as in daily steps), maternal fruit and vegetable intake and serving animal protein to their children. 50-min Focus Group Discussion (FGD) on how to overcome barriers towards child feeding practices followed by 30 min creating a pledge and strategies for tackling stunted child and overweight/obese mother (SCOWT) from the mother’s own perspective.
Vicarious experience	Watching video of how others perform responsive feeding, maternal self-efficacy for preparing more animal protein will improve.	Session 5	50-min nutrition education class on child feeding practices with emphasis on responsive feeding.
Verbal Motivation	Verbal motivation from a respected informal leader such as community health worker will improve maternal self-efficacy for: 1. Healthy grocery shopping 2. Food preparation 3. Physical activity	Home visit Session 6	The motivational interviewing delivered through home visit (six times throughout the study administered on alternate weeks from nutrition education class and hands-on activity sessions) will focus on providing verbal motivation for mothers to achieve their biweekly goals (consisting of improving daily steps, increasing maternal fruit and vegetable intake, and serving their child animal protein), and help with strategies to overcome barriers. 30-min of role playing and jingle/song making related to combating child stunting.

The nutrition education classes were administered by three investigators in Indonesia with expertise in nutrition and behavioral change intervention who are on the faculty of the Department of Nutrition, Universitas Airlangga. All of the investigators hold a master’s degree in public health or community nutrition. The hands-on experience session was delivered by two trained research assistants who each hold a bachelor’s degree in public health nutrition.

The motivational interviewing was delivered through home visits, which took place six times throughout the study, administered on alternate weeks from nutrition education class and hands-on activity session. The visits focused on providing verbal motivation for mothers to achieve their biweekly goals, which centered on improving daily steps, fruit and vegetable intake, and serving child meals with animal protein, and helping them with strategies to overcome barriers. Each motivational interviewing session lasted for approximately 1 h. The motivational interviewing session took place during a home visit and was delivered by a trained community health worker living in the participant’s area.

### Comparison group: usual care plus print educational materials

The comparison group did not receive nutrition education or home visits by a community health worker. Participants in the comparison group received sets of printed educational materials, coupled with benefits from a government food supplementation program. The sets of printed educational materials (booklets) were provided after baseline data measurement for both intervention and comparison group. Printed material was chosen as the medium for source of information due to several considerations: to mimic government strategy for distributing the health messages through the “Kartu Menuju Sehat” (KMS) or a child’s growth monitoring card and because it was deemed unethical not to provide anything for participants knowing they had double burden of malnutrition in the form of SCOWT. Finally, these materials were provided to minimize the threat to internal validity that might occur. By providing the comparison group with the same printed educational materials, we aimed to avoid resentful demoralization of participants in the comparison group. All mothers participating in the study received usual care in the form of monthly growth monitoring of their child’s nutritional status at *posyandu* and access to a supplementary feeding program from Indonesian government. Supplementary feeding program was given for children under 5 years old that consist of foods equivalent with 1000 to 1550 kcal total energy and 25–39 g protein [22].

### Primary outcome

Outcome measurements were taken at baseline and at the end of the study (after three months). The primary outcome, on which power was calculated, is the change in child’s height (cm). Details of height measurement are found within the anthropometric data section below. The same methods of height assessment were used at baseline and subsequent measurement periods.

### Secondary outcomes

Secondary outcomes for this study consist of variables from anthropometric data, lifestyle data, and psychological data.

#### Anthropometric data

The secondary outcome measured in this study was maternal overweight/obesity and child stunting or the double burden of malnutrition measured as SCOWT. Data were collected on child’s age, weight and height, as well as maternal weight, height, and waist circumference. Weight was measured in light clothing, without shoes or sandals, on a Camry EB6571 digital scale (Guangdong, China) to 0.01 kg for weight. Height was measured to the nearest 0.1 cm using a stadiometer (SECA 213). Maternal weight and height measurement were used to calculate BMI. Waist circumference was measured horizontally halfway between the lowest rib and the upper prominence of the pelvis using a non-extensible steel tape (MyoTape) placed against the bare abdomen. We measured the change in maternal body mass index (BMI) and child height for age z-score (HAZ) as the secondary health outcome using the 2005 WHO reference standard to assess maternal and child nutritional status. All measurement will be done and recorded twice to ensure the validity. The third measurement will be taken if the difference between the prior two measurement differ by more than 1 %.

#### Lifestyle data

A household dietary diversity questionnaire was used to estimate maternal fruit and vegetable intake as well as the child’s animal protein intake [23]. The 24-h dietary recall was completed for two days, with one day in between the two. The secondary variables measured were total energy intake, total protein intake and total fat intake for the mother. The 24-h recall interviews were performed by research assistants. They were trained before the interview to follow a standardized protocol to ask neutral probing questions to encourage recall of food items and to teach different methods of food preparation and brands available in different cultures. Dietary data were analyzed using food processor software drawing from a database of Indonesian Food updated yearly by the Department of Nutrition, *Universitas Airlangga* (UA) – Indonesia. Actual spending of the voucher was evaluated in collaboration with the local grocery store recording each participant’s grocery shopping behavior. Maternal physical activity was measured in total daily steps using the Yamax Digiwalker Pedometer SW200 (Tokyo, Japan). Even though pedometer was less robust than accelerometer, this device is still regarded as an objective measure of physical activity in the previous study [24]. Mothers wore the pedometer for three consecutive days from each time point of measurement at baseline, 3 months, 6 months and 12 months evaluation. We generated a wear-time log for each mother to estimate time worn to arrive at a true daily step count.

#### Psychological variables

Outcome expectations about the targeted behavior of maternal fruit and vegetable intake as well as maternal physical activity and animal protein fed to their child were measured using the 30-item questionnaire adapted from several sources [25–27]. Sedentary behavior and sitting time were assessed using the last 7-d sedentary behavior questionnaire (SIT-Q-7d) [28]. Maternal self-efficacy to engage in physical activity was measured using a 10-item questionnaire on barriers to self-efficacy and an 8-item questionnaire on self-efficacy of performing the task. Maternal self-efficacy toward fruit consumption was measured using a 6-item questionnaire on barriers to self-efficacy and a 6-item questionnaire on performing the fruit consumption task. Maternal self-efficacy toward vegetable consumption was measured using an 8-item questionnaire on barriers to self-efficacy and an 8-item questionnaire on performing the vegetable consumption task. Maternal self-efficacy in serving and feeding their child animal protein will be measured using a 10-item questionnaire on barriers to self-efficacy and a 15-item questionnaire on performing the task of serving and feeding their child animal protein. All of the self-efficacy questionnaires were developed as Likert scale answers based on Bandura’s guide for constructing self-efficacy scales [29]. For all psychological variable we tested the Cronbach alpha prior to data analysis to test the internal consistency.

#### Moderating variables

Moderating variables such as age, child’s gender, family size, occupational status, educational attainment, nutritional literacy and food insecurity were collected through structured face-to- face interviews at the baseline. Number of children, household income, and food expenditure were used to estimate socioeconomic status (SES). Food insecurity was measured using the Household Food Insecurity Access Scale (HFIAS) guidelines [30].

### Process evaluation

To provide a sense of the quality of the outcomes to be measured, we conducted several measures of “process evaluation”. First, we monitored adherence to the intervention by making a log of nutrition education sessions including an attendance list filled by our research assistants in the form of biweekly goal-setting sheets completed by mothers at the end of class and following hands-on activity sessions. Through collaboration with the local grocery store, we monitored maternal grocery purchases from the recorded grocery voucher given to mothers each time they attended the class and hands-on session. In addition, a log of daily steps was recorded as a part of the motivational interviewing session with the community health worker during the home visit. Fidelity to the messages and curriculum was monitored by our research assistant that presented at the nutrition education classes, and also from the verbal report and feedback given by the community health workers to our research assistant after the home visit.

### Statistical analysis plan

We conducted a meditational analysis to examine whether changes in behavioral and psychological factors of the SCT construct (self-efficacy, outcome expectation and goal setting) mediated the association between the intervention and outcomes. Subgroup analysis was used to examine whether the association between the intervention and the outcomes were modified by socioeconomic status (SES), and demographics measured at baseline, such as age, number of children, educational status, employment, and nutritional literacy.

For all variables that are normally distributed or transformed to normality, we analyzed the difference in the outcome between the control and intervention group using a mixed factorial ANOVA. We used the household food insecurity access scale (HFIAS) score as covariates in the analysis. Furthermore, we conducted the ANCOVA test to see the difference in changes of primary and secondary outcomes adjusted for baseline value and the HFIAS score. For nonparametric statistics, we employed the two related-samples Mann Whitney U test and analyzed the data separately for the NEO-MOM group and the PRINT group with Bonferroni correction. All data analyses were performed in IBM SPSS Statistics 22 (Armonk, NY). The statistical significance for all tests was set at an alpha level of 0.05. In the event that after a follow-up of the intervention, the study has high noncompliance or missing outcomes, we planned to conduct an intention-to-treat (ITT) analysis.

## Discussion

This study compared the effectiveness of the NEO-MOM intervention with usual care plus printed educational materials primarily for increasing child’s height, but secondarily for decreasing child stunting and maternal overweight and obesity. The study setting is an urban population in Surabaya City, Indonesia. It was hypothesized that participants in the intervention group would benefit from verbal motivation given by a community health worker during biweekly home visits, and that this approach would magnify the effect of the six sessions of behaviorally oriented nutrition education.

One strength of the study is that the setting includes the infrastructure for dissemination of research finding through *posyandu* and the possibility of reaching a large number urban inhabitants of Surabaya City. A limitation is the fact that variation and inequalities between the islands of Indonesia cannot be captured. Because Surabaya City is the center of urbanization, and second only to Jakarta, ethnicity and deprivation have been addressed in the study design. Other limitations include use of self-reported data (e.g., 24 h food recall) and imprecise estimates of physical activity through use of pedometers.

The intervention suggests actions to support behavior change, such as improved self-efficacy, biweekly goal setting and verbal motivation delivered through home visits. We employed anthropometric measurements of mother-child pairs three times during recruitment to reduce measurement bias. Selection bias was reduced by random allocation of consenting participants. However, some degree of selection bias might have been introduced as consenting participants might already have been motivated to join the study. Because of the nature of the intervention, blinding participants and research assistants delivering the intervention was not possible. Objective anthropometric measurements, use of a blinded assessor for baseline and post-intervention data collection, as well as a standardized procedure for self-reports were applied to limit potential bias during outcome assessment.

## Abbreviations

ANOVA, analysis of variance; ANZCTR: Australian New Zealand Clinical Trials Registry; BMI, body mass index; FGD, Focus Group Discussion; HAZ, height-for-age z-score; ICMJE, International Committee of Medical Journal Editors; IRB, Institutional Review Board; ITT, intention-to-treat; NCD, non-communicable disease; NEO-MOM, Nutrition Education for Overweight/Obese Mother with Stunted Children; SCOWT, stunted child and overweight/obese mother; SCT, Social Cognitive Theory; SES, socio economic status; SIT-Q-7d, the last 7-d sedentary behavior questionnaire; SUN, scaling-up nutrition; UTN, Universal Trial Number; WHO, World Health Organization
